# Social and Environmental Determinants of Childhood Stunting in Indonesia: National Cross-Sectional Study

**DOI:** 10.2196/68918

**Published:** 2025-10-10

**Authors:** Yuni Sufyanti Arief, Fildzah Cindra Yunita, Ferry Efendi, Fadhaa Aditya Kautsar Murti, Rifky Octavia Pradipta, Lisa McKenna

**Affiliations:** 1 Department of Fundamental Nursing, Faculty of Nursing, Universitas Airlangga, Jl Mulyorejo, Campus C Universitas Airlangga, Surabaya, 60115, Indonesia, 62 8123106365; 2 Research Group for Health and Wellbeing of Women and Children, Faculty of Public Health, Universitas Airlangga, Banyuwangi, Indonesia; 3 Department of Advanced Nursing, Faculty of Nursing, Universitas Airlangga, Surabaya, Indonesia; 4 School of Nursing and Midwifery, La Trobe University, Melbourne, Australia; 5 Research Center in Advancing Community Healthcare (REACH), Universitas Airlangga, Surabaya, Indonesia; 6 Department of Biostatistics and Population Studies, Faculty of Public Health, Universitas Indonesia, Depok, Indonesia

**Keywords:** associated factors, stunting, children under five years, social determinants of health, children

## Abstract

**Background:**

The cause-effect of stunting is known as a complex factor, including family, environmental, social, and cultural factors, in stunting among children. Yet, the latest updated associated factors emphasizing on social and environmental factors are still limited.

**Objective:**

This study aimed to analyze the latest evidence on the factors associated with stunting, with a particular focus on various factors.

**Methods:**

A secondary data analysis using the 2023 Indonesia Health Survey (Survei Kesehatan Indonesia [SKI] 2023) was conducted. This study analyzed a total of 78,049 (or 81,068 if weighted) children aged 5 years and younger who had a complete response to all interest variables. Bivariate analysis using the Pearson *χ*^2^ test with a *P* value of <.05 for determining a significant association and a multivariate analysis for further analysis of the association between the outcome and each predictor were implemented.

**Results:**

The prevalence of stunting in this study was 15,958/78,049 children (19.69%). In the adjusted analysis, immunization status (adjusted odds ratio [aOR] 1.34, 95% CI 1.22‐1.48; *P*<.001) and KPS (Kartu Perlindungan Sosial; Social Protection Card) ownership (aOR 1.13, 95% CI 1.05‐1.21; *P*<.001) were significantly associated with higher odds of stunting. Conversely, children in the wealthiest quintile were significantly less likely to experience stunting compared to those from the poorest families (aOR 0.47, 95% CI 0.42‐0.52; *P*<.001). Other variables, such as household water sources (aOR 1.18, 95% CI 1.00‐1.37; *P*=.04), and geographical location, particularly in Sulawesi (aOR 1.23, 95% CI 1.14‐1.33; *P*<.001) and Papua and Maluku (aOR 1.20, 95% CI 1.08‐1.33; *P*<.001), were also significantly associated with increased odds of stunting.

**Conclusion:**

Not receiving immunization, consuming water from unimproved sources, ownership of a Social Protection Card, and living in regions such as eastern Indonesia were significantly associated with childhood stunting. These findings emphasize that social and environmental factors remain critical determinants of stunting. Improving multifaceted and holistic interventions, with a focus on immunization coverage, good water access, social protection, and reducing regional disparities, is essential to accelerate progress toward stunting reduction targets.

## Introduction

Stunting, defined as poor linear growth with a height-for-age z-score less than –2 SDs of the median determined in the World Health Organization’s (WHO) Child Growth Standards [1], is still a global public health issue, especially in low- and middle-income countries [2-4]. The condition has been known as a significant indicator of chronic malnutrition and leaves irreversible impediments throughout life [5,6]. Approximately, 148.1 million (22.3%) of children aged 5 years and younger were diagnosed as stunted worldwide in 2022 [4], with an estimated 98% children with stunting residing in low- and middle-income countries [7] and about 52% living in Asia [4]. The consequences of stunting can reflect on further child development, including in terms of health, physical and cognitive, leading to a higher risk of morbidity and mortality [8-10].

According to the Indonesia Health Survey (Survei Kesehatan Indonesia, SKI) in 2023, the prevalence of stunting in Indonesia remains substantial at 21.5%, indicating that about 1 in 5 children ages 5 years and younger is affected, with the highest incidence observed among children aged 24 to 35 months [11]. Digging deeper to the geographical location, huge geographical disparities of children with stunting exist in which eastern regions of Indonesia, namely Papua, Nusa Tenggara, and Sulawesi, indicated a significantly higher prevalence [12]. This percentage of children showed a steady decrease in the past decade [13], yet the number is still far from Indonesia’s national target in reducing child stunting to 14% by 2024 [14]. Furthermore, the Joint Malnutrition Estimates (JME) released in 2023 recorded that only one-third of all countries worldwide are on track to cut across half the percentage of children affected by stunting [4]. Reflecting on this evidence, a comprehensive evaluation of existing stunting reduction programs and a more in-depth analysis of the associated factors is needed.

The etiology of stunting is multifactorial. The WHO developed the conceptual framework on childhood stunting [15], explaining the cause-effect of complex factors, including family, environmental, social, and cultural factors, in stunting among children aged 5 years and younger. Previous studies have also justified that maternal factors, such as mother’s height, mother’s BMI, antenatal care history, mother’s education, are considered as another direct factor of stunting among children after the child’s factors [2,5,16-18]. While the children-related factors that have been discovered include the child’s age, sex, low birth weight, feeding practice, exclusive breastfeeding, lack of food intake, and infection or disease history [5,16,19,20]. Beyond biological and behavioral factors, a growing body of research highlights the importance of socioeconomic [2,18] and environmental conditions, including household wealth, access to clean water, sanitation, hygiene practices, and geographic disparities, as indirect but critical determinants of stunting [17,21-23].

Despite these known pathways, there remains limited large-scale national evidence from Indonesia that focuses specifically on how social and environmental (or exposome) factors influence stunting outcomes. Given Indonesia’s vast geographic diversity and persistent regional inequalities, it is essential to examine how broader contextual factors interact with stunting risks. This study aims to fill this gap by analyzing nationally representative data to identify key social and environmental determinants of stunting among Indonesian children ages 5 years and younger. The findings will provide actionable insights for designing more context-specific, equity-oriented interventions and contribute to accelerating national progress toward the 2024 target and Sustainable Development Goals (SDGs).

## Methods

### Study Design and Data Collection

A secondary data analysis was conducted by extracting data from the 2023 Indonesia Health Survey (Survey Kesehatan Indonesia) for which the survey is the integration of 3 researches: the Basic Health Research (Riset Kesehatan Dasar, RISKESDAS), the Indonesia Nutritional Status Monitoring Survey (Survei Status Gizi Indonesia, SSGI), and Biomedical and dental-and-oral examinations. The survey had included a nationally representative sample of households, households with children aged 5 years and younger, and individuals’ characteristics with a total sample of 877,531 household respondents and 314,161 households with children aged 5 years and younger, from 38 provinces and 514 administrative cities or districts across Indonesia.

### Sampling Technique and Study Population

In the SKI 2023, stratified sampling technique was used at the block level census and household level of chosen block level census, to get a representative sample for all provinces. A total of 34,065 blocks census was stratified using explicit stratification; meanwhile, implicit stratification was applied for the household level. The response rate of SKI 2023 was 98.74% with 1,191,692 respondents, consisting of interviewing 877,531 respondents of member of households and anthropometry examination of 84,267 respondents of children aged 5 years and younger (see Multimedia Appendix 1). Before collecting data, informed consent was obtained from every respondent. This study analyzed a total of 78,049 children (or 81,068 if weighted) aged 5 years and younger who had a complete response to all interest variables (sex, immunization status, last months’ history of diarrhea, place of residence, social protection card ownership, household water sources, water quality, and geographical location). Weight and sampling design were used for adjustments in this analysis.

### Data Collection and Procedure

The SKI 2023 took place from August 2023 to early October 2023. There were 3 questionnaires used in this survey, namely: household questionnaire, children aged 5 years and younger questionnaire, and individual questionnaire, all of which reflect the population and health-related issues relevant to Indonesia. This study used the children’s data for analysis. Data were requested legally to the Ministry of Health website [24]

### Measurement of Stunting and Covariates

The stunting among children aged 5 years and younger was the dependent variable of interest, while several independent variables like socioeconomics and environments were included in the analysis depending on the availability in SKI 2023. Stunting was defined using HAZ (height-for-age z-score), considering the sex, height, weight, and age of individuals. Individuals with a z-score< –2 were categorized as stunted. Our definition matched with the standard defined by the WHO [25].

Water quality was categorized as poor and good, meanwhile household water source was categorized as improved, unimproved, and surface water. Water quality was defined by the physical parameters of the water. If a household responded “No” to all these physical quality issues (turbidity, color, taste, foam, and odor), their drinking water was categorized as good quality [26]. Improved water sources consisted of packaged water, refilled water, piped water, boreholes or wells with pumps, protected dug wells, protected springs, rainwater collection, water hydrants, water bought from vendors, and water terminals. Unimproved water sources included unprotected dug wells and unprotected springs. Surface water, such as water from rivers, lakes, or irrigation canals, was classified separately. This classification was derived from the Demographic and Health Surveys (DHS) categorization [27].

Last months’ history of diarrhea was categorized as yes, no, and don’t know. This 3-category classification was used because recall of diarrheal episodes over the previous month can be subjective and uncertain. Respondents may not clearly remember or recognize whether symptoms met the criteria for diarrhea, so the “don’t know” option captures this uncertainty. For immunization status, the categorization was divided as yes and no or unsure. Immunization records tend to be more definitive, as caregivers often have vaccination books (KIA [Kesehatan Ibu dan Anak], the Mother Child Health Book, or the pink book) to clearly recall vaccination history. Thus, grouping answers to “no” and “unsure” simplifies analysis by treating any lack of confirmed vaccination as absence of immunization. Female and male were a categorization used for sex. Place of residence was categorized as rural and urban. Social Protection Card (Kartu Perlindungan Sosial, KPS) ownership was categorized as yes and no. Geographical location of children was categorized as Java, Sumatera, Bali and Nusa Tenggara, Kalimantan, Sulawesi, and Papua and Maluku. In this study, the wealthy index was grouped into 5 quintiles as poorest, poor, middle class, wealthier, and wealthiest. Prevalence of stunting was calculated for each category of the covariates to assess distribution patterns.

### Statistical Analysis

Initial analysis started with frequency distribution analysis of the sociodemographic characteristics and relevant external factors associated with stunting among children. To assess the frequency distribution, bivariate analysis using Pearson *χ*^2^ test was implemented with a *P* value of <.05 for determining a significant association. The association between outcomes and predictor variables was analyzed using binary logistic regression as presented in a crude or unadjusted analysis and to examine the association between predictors and stunting. Odds ratios (OR) with 95% CI were reported.

The selection of reference categories was selected based on findings from previous research, which indicate that the risk of stunting tends to increase among children with incomplete immunization, recent episodes of diarrhea, lower wealth quintile status, and those residing in rural areas [28-31]. In line with these findings, the reference groups were chosen to allow meaningful comparison. Finally, a multivariate analysis has been done to assess further association between the outcome and each predictor. All statistical analysis was implemented using SPSS (version 26.0 for Windows; IBM Corp).

### Ethical Considerations

This study used secondary, deidentified data from the 2023 Indonesian Health Survey (SKI 2023), which was conducted by the Indonesian Ministry of Health with previous approval from the National Ethics Committee. As this study used publicly available, anonymized data, it was classified as exempt from additional ethics review. The National Ethics Commission classified this study as “exempted” and the Indonesian Ministry of Health collected the data with informed consent, ensuring participants signed a consent form, emphasizing that participation was voluntary and confidential. The Indonesian Ministry of Health has made the data available to the academic community through its website [24].

## Results

### Prevalence of Stunting Among Covariates

The prevalence of stunting in this study was 15,958/78,049 (19.69%). Among the water and sanitation factors, the prevalence of stunting was dominant in households with poor water quality 665 (21.9%). In addition, households with surface water sources had higher prevalence of stunting 96 (29%) compared to unimproved 636 (25.2%) and improved water sources 15,226 (19.5%).

Among individual factors, we found that the prevalence of stunting was high among children who had diarrhea last month 968 (24.7%) and also male children 8601 (20.8%). Children with no status of vaccination were also seen with higher prevalence of stunting 1593 (25%). In terms of community factors, we found that children who lived in rural areas had a higher prevalence of stunting 7266 (21.7%). In addition, households who had KPS had a higher prevalence of stunting 3439 (23.4%) and also households in the poorest, poorer, and middle class had higher prevalence of stunting 2800 (27.6%), 3395 (23.5%), and 3488 (20.5%), respectively. The prevalence of stunting was also higher in Papua and Maluku 601 (26.2%), Sulawesi 1538 (24.7%), and Kalimantan 1039 (20.5%; see Table 1).

**Table 1.** Prevalence of stunting among covariates.

Variables		Prevalence of stunting, n/N (%)
Water quality		
Poor		665/3042 (21.9)
Good		15,293/78,026 (19.6)
Household water sources		
Improved		15,226/78,219 (19.5)
Unimproved		636/2525 (25.2)
Surface water		96/325 (29.6)
Last months’ history of diarrhea		
No		14,955/77,038 (19.4)
Yes		968/3920 (24.7)
Don’t know		35/110 (31.6)
Sex		
Female		7358/39,752 (18.5)
Male		8600/41,317 (20.8)
Immunization status		
Yes		14,365/74,703 (19.2)
No or unsure		1593/6365 (25)
Place of residence		
Rural		7266/33,548 (21.7)
Urban		8693/47,520 (18.3)
KPS**^a^** ownership		
No		12,519/66,375 (18.9)
Yes		3439/14,693 (23.4)
Geographical Location		
Java island		8171/43,070 (19)
Sumatera		3556/19,481 (18.3)
Bali and Nusa Tenggara		1053/4914 (21.4)
Kalimantan		1039/5072 (20.5)
Sulawesi		1538/6233 (24.7)
Papua and Maluku		601/2297 (26.2)
Wealth quintile		
Poorest		2800/10,147 (27.6)
Poorer		3395/14,459 (23.5)
Middle class		3488/16,994 (20.5)
Wealthier		3432/19,296 (17.8)
Wealthiest		2843/20,172 (14.1)

a KPS : Kartu Perlindungan Sosial (Social Protection Card).

### Factors Associated With Stunting

In the bivariate analysis with the unadjusted model (see Table 2), we found that water quality, household water sources, immunization status, KPS ownership, and also wealth quintile were associated with stunting (*P* values<.05). Both geographical and history of diarrhea were partially associated with stunting, as only some categories were significant with stunting (*P* values<.05). However, some independent associations in bivariate analysis were not strong enough to be significant after adjusting with other variables.

**Table 2.** Factors associated with stunting among children aged 5 years and younger.

Variables		Unadjusted		Adjusted	
		*P* value	OR^a^ (95% CI)	*P* value	aOR^b^ (95% CI)
Water quality					
Not good		Ref^c^	Ref	Ref	Ref
Good		.046	0.87 (0.76-0.99)	.82	0.98 (0.85-1.12)
Household water sources					
Improved		Ref	Ref	Ref	Ref
Unimproved		<.001	1.39 (1.19-1.62)	.04	1.18 (1.00-1.37)
Surface water		<.001	1.74 (1.28-2.36)	.11	1.29 (0.94-1.76)
Last months’ history of diarrhea					
Don’t know		Ref	Ref	Ref	Ref
Yes		.08	0.52 (0.25-1.08)	.41	0.72 (0.33-1.56)
No		.40	0.71 (0.33-1.49)	.11	0.54 (0.25-1.15)
Sex					
Female		Ref	Ref	Ref	Ref
Male		<.001	1.15 (1.09-1.22)	<.001	1.16 (1.09-1.22)
Immunization status					
Yes		Ref	Ref	Ref	Ref
No or unsure		<.001	1.40 (1.28-1.52)	<.001	1.34 (1.22-1.48)
Place of residence					
Rural		Ref	Ref	Ref	Ref
Urban		<.001	0.80 (0.76-0.85)	.40	0.97 (0.91-1.03)
KPS^d^ ownership					
No		Ref	Ref	Ref	Ref
Yes		<.001	1.31 (1.22-1.40)	<.001	1.13 (1.05-1.21)
Geographical location					
Java island		Ref	Ref	Ref	Ref
Sumatera		.17	0.95 (0.89-1.02)	<.001	0.84 (0.79-0.91)
Bali and Nusa Tenggara		.001	1.16 (1.06-1.27)	.94	0.99 (0.91-1.09)
Kalimantan		.03	1.09 (1.00-1.20)	.20	1.06 (0.96-1.16)
Sulawesi		<.001	1.39 (1.29-1.50)	<.001	1.23 (1.14-1.33)
Papua and Maluku		<.001	1.51 (1.37-1.66)	<.001	1.20 (1.08-1.33)
Wealth quintile					
Poorest		Ref	Ref	Ref	Ref
Poorer		<.001	0.80 (0.73-0.88)	<.001	0.83 (0.76-0.91)
Middle class		<.001	0.67 (0.62-0.73)	<.001	0.71 (0.65-0.78)
Wealthier		<.001	0.56 (0.51-0.62)	<.001	0.61 (0.55-0.67)
Wealthiest		<.001	0.43 (0.39-0.47)	<.001	0.47 (0.42-0.52)

a OR: odds ratio.

b aOR: adjusted odds ratio.

c Ref: Reference.

d KPS: Kartu Perlindungan Sosial (Social Protection Card).

In the adjusted models (see Table 2), the variables that remained significant were immunization status, KPS ownership, and wealth quintile. We found that children with no immunization status had higher odds of developing stunting (aOR 1.34, 95% CI 1.22-1.48). In addition, children whose households owned KPS also had higher odds of developing stunting (aOR 1.13, 95% CI 1.05-1.21). We also noticed a gradient of decreasing risk along with the increasing status of wealth quintile. Children in the wealthiest (aOR 0.47, 95% CI 0.42-0.52), wealthier (aOR 0.61, 95% CI 0.55-0.67), middle (aOR 0.71, 95% CI 0.65-0.78), and poorer (aOR 0.83, 95% CI 0.76-0.91) classes had lower odds of developing stunting compared to children in the poorest class.

Partial associations in the adjusted model were found in the variables such as household water sources and geographical location (see Table 2). Children whose household water was unimproved had higher odds of developing stunting (aOR 1.18, 95% CI 1.00-1.37), but such association was not found in the surface water categories. Finally, children who lived in Sulawesi (aOR 1.23, 95% CI 1.14-1.33), and Papua and Maluku (aOR 1.20, 95% CI 1.08-1.33) had higher odds of developing stunting compared to children who lived in Java, but the odds of stunting were lower for children in Sumatera (aOR 0.84, 95% CI 0.79-0.91). Such an association was not found in Bali, Nusa Tenggara, and Kalimantan.

## Discussion

### Principal Findings

In this study, the prevalence of stunting among children aged 5 years and younger was 19.69% (15,958/78,049). Although this figure appears lower than the national estimate reported by SKI 2023, which recorded a prevalence of 21.5% [11], it is important to note that our analysis used a different subset of the same dataset. Specifically, the discrepancy may be due to the application of distinct inclusion criteria in our study that focused on children with complete data for selected socioeconomic and environmental variables. In which, we may have excluded some high-risk groups or regions. Therefore, this finding should not be interpreted as a trend or decline in stunting prevalence over time, but rather as a context-specific estimate based on an analytical sample tailored to explore social and environmental determinants.

A downward trend in stunting prevalence has highlighted an updated figure in this study. Comparing to the overall report of SKI 2023, the existence of variation in the prevalence of stunting, even though using the same dataset, might be attributable to differences in the inclusion criteria for the variable use. As this study includes the set of data on influential factors, more of which are social and environmental factors. This finding is lower than the findings from India (31.7%) [32], Afghanistan (44.7%) [33], Bangladesh (26.7%) [34], Pakistan (40%) [35], and Timor Leste (45.1%) [4]. The difference in findings might rely on the efforts in public health intervention, such as nutritional programs, economic and social development conditions, accessible community-based health services, environmental focus, and the disparities of investment in nutritional-sensitive strategies. Previous studies [36,37] revealed that inadequate investment in nutrition could hinder the progress of reducing stunting to reach the global goals of 40% by 2030. Hence, the positive result of Indonesia’s integrated efforts should be in scaling up existing programs and investing more in nutrition-sensitive programs.

This study revealed that children who did not receive immunization are 1.34 times higher odds of being stunted compared to children who received immunization. Some studies are in line with findings across the literature [19,38-42]. This association could be due to interrelated factors, particularly infection rates and nutritional status. Inadequate immunization is linked to the high risk of infectious diseases, which can negatively affect a child’s growth and development [43,44]. In addition to direct impact on nutritional status, a study has proven the significant improvement of anthropometric outcomes among children who have received immunization [45]. At this point, addressing stunting requires a comprehensive programmatic approach, integrating immunization programs with maternal and child health, rather than relying solely on nutritional intervention programs.

Our study found that children from households receiving KPS were significantly more likely to be stunted compared to those not receiving the benefit. This is in line with a previous study that mentioned that the Conditional Cash Transfer program does not result in reducing stunting in poor children aged 5 years and younger [46]. On the other side, children of household-owned KPS are significantly associated with stunting, which is an unexpected result, as KPS are originally designed to alleviate poverty and improve access to basic needs, including food, health care, and education. Several potential mechanisms may explain this association. First, the presence of KPS itself is an indicator of household vulnerability. The families receiving it are among the poorest, and the underlying socioeconomic disadvantages may outweigh the benefits received from KPS. Second, while the KPS provides rice subsidies, school allowances, and limited health support, it may not directly address dietary diversity, child feeding practices, or access to quality health care, which are critical determinants of nutritional status. Third, there may be issues related to implementation or adequacy. A study in Palembang, Indonesia, mentions that families with more than 4 members or more, the children have the risk of stunting even if they already get subsidized [47]. The value of support provided may be insufficient to meet a household’s nutritional needs. Finally, some households may not use the benefits effectively due to lack of nutrition literacy or limited access to supporting services.

The study found that as the wealth quintile increases, the risk of stunting among children aged 5 years and younger decreases, with children from the wealthiest families being less likely to be stunted than those from the poorest families. Previous studies have proven the same result, that is, children from the poorest families are prone to develop stunting [2,3,16,18]. The relationship might be attributable to economic inequality between quintile groups, where children from wealthiest families often have food security, accessibility to consume a high quality of foods, health care services, improved sanitation, and water resources [48,49]. On the other hand, all these facilities are being a limit for children from the poorest families [18]. Nutrition intervention [50-52], such as providing supplementary foods for children with stunting, has been implemented by the Indonesian Government and showed a significant result in intervening undernutrition issues [51]. Expanding these interventions across Indonesia, with an emphasis on equal access in all regions, is essential for addressing stunting on a larger scale.

In this study, children exposed to unimproved water sources had 1.18 times higher odds of being stunted compared to children who were exposed to improved water sources. A study in Ethiopia, India, Peru, and Vietnam is correspondent with this finding, which showed the consumption of improved water source decreasing the risk of stunting among children [19,53]. Consuming from an unimproved water source increases the infection rate and the incidence of diarrhea among children that indirectly affects nutritional status of children [54]. Unimproved water sources might contain pathogenic microorganisms and other chemical substances that cause diarrhea or other infectious diseases [22]. Some studies justified that intervention on hygiene and sanitation is significantly improving children’s growth [55-57], indicating sanitation and hygiene improved water sources intervention must be included in the national program for achieving the stunting reduction target.

In terms of geographical location, the odds of being stunted among children who reside in Sulawesi and Papua and Maluku are 1.23 times and 1.20 times, respectively, compared to those who reside in Java. Meanwhile, children living in Sumatera are less likely to be stunted compared to children living in Java. Common findings also have been proven by studies that tried to map the stunting prevalence in and across Indonesia [12,18]. The rationale is exacerbated by inequality conditions across Eastern Indonesia, of which many regions have limited access to decent health facilities and resources [58,59], particularly the proportion of health care personnel and services as they are centralized in Java and Sumatera. These findings suggest the government should enhance the infrastructure developments, especially in health, and distribute more health personnel outside Java and Sumatera Island.

The findings from this study offer several implications for policy and program planning. First, the identification of social and environmental determinants, such as low household wealth, regional disparities, poor access to clean water, and incomplete immunization. This highlights the need for multisectoral approaches that extend beyond direct nutrition interventions. Nutrition programs should be designed with a broader lens, integrating improvements in public health infrastructure, social protection targeting, sanitation, and health education. For example, linking immunization programs with nutritional screening and counseling may enhance early identification of at-risk children. Second, the geographic disparities observed support the prioritization of region-specific interventions, particularly in Papua, Maluku, and Sulawesi, where stunting remains persistently high. Tailoring intervention packages based on local infrastructure, culture, and needs can increase effectiveness. Finally, the unexpected finding regarding KPS beneficiaries emphasizes the importance of monitoring and evaluating the impact of social programs on nutrition outcomes and modifying them to include behavior change communication, food diversification support, or conditional nutrition services. In this way, evidence from large-scale secondary datasets can directly inform data-driven, equity-focused strategies to reduce stunting nationally.

### Strengths and Limitations of the Study

A large dataset as representative of the national population of Indonesia has been used in this study. However, the researcher has limited control over the selection variables, data quality, and indicators for measurements due to the use of secondary dataset. Social desirability bias might exist since the survey used self-reports for gathering the information. Some potentially important determinants of stunting, such as detailed dietary intake, feeding practices, or maternal nutritional status, were not available, and therefore could not be included. Second, the use of self-reported data introduces the possibility of social desirability and recall bias, which may affect the accuracy of responses, particularly in sensitive areas such as household wealth or immunization history. Another limitation is the cross-sectional design of this study, which allows for the identification of correlations between exposures and outcomes but does not permit determination of the temporal sequence of events. As a result, causal inferences about the relationship between the identified risk factors and stunting cannot be made.

### Conclusion

This study reinforces the idea that childhood stunting remains a significant public health concern requiring multifaceted interventions. Key social and environmental factors, including incomplete immunization, KPS ownership, low household wealth, use of unimproved water sources, and residence in Sulawesi, Papua, and Maluku were all significantly associated with the increased odds of stunting. These findings offer updated national evidence on the contextual determinants of stunting in Indonesia.

To accelerate progress in stunting reduction, increasing immunization coverage should be prioritized, supported by community-based health education targeting mothers and caregivers. The association between KPS recipients and higher stunting risk suggests that existing welfare programs may need to be strengthened or better integrated with nutrition-sensitive interventions. Furthermore, sanitation factors, particularly access to improved water sources, should receive greater policy attention. Finally, improving access to quality health care across all regions, especially underserved areas, is essential for effective and equitable stunting prevention efforts nationwide.

## Supplementary material

10.2196/68918 Multimedia Appendix 1 Sampling technique.

## Abbreviation

KPS: Kartu Perlindungan Sosial (Social Protection Card)
SKI: Survei Kesehatan Indonesia (Indonesia Health Survey)
WHO: World Health Organization
