# Supplementary figures and images for: Social and Environmental Determinants of Childhood Stunting in Indonesia: National Cross-Sectional Study
Source: JMIR Pediatr Parent. 2025 Oct 10;8:e68918. doi: 10.2196/68918 (PMC12527368; doi:10.2196/68918)

Sampling technique


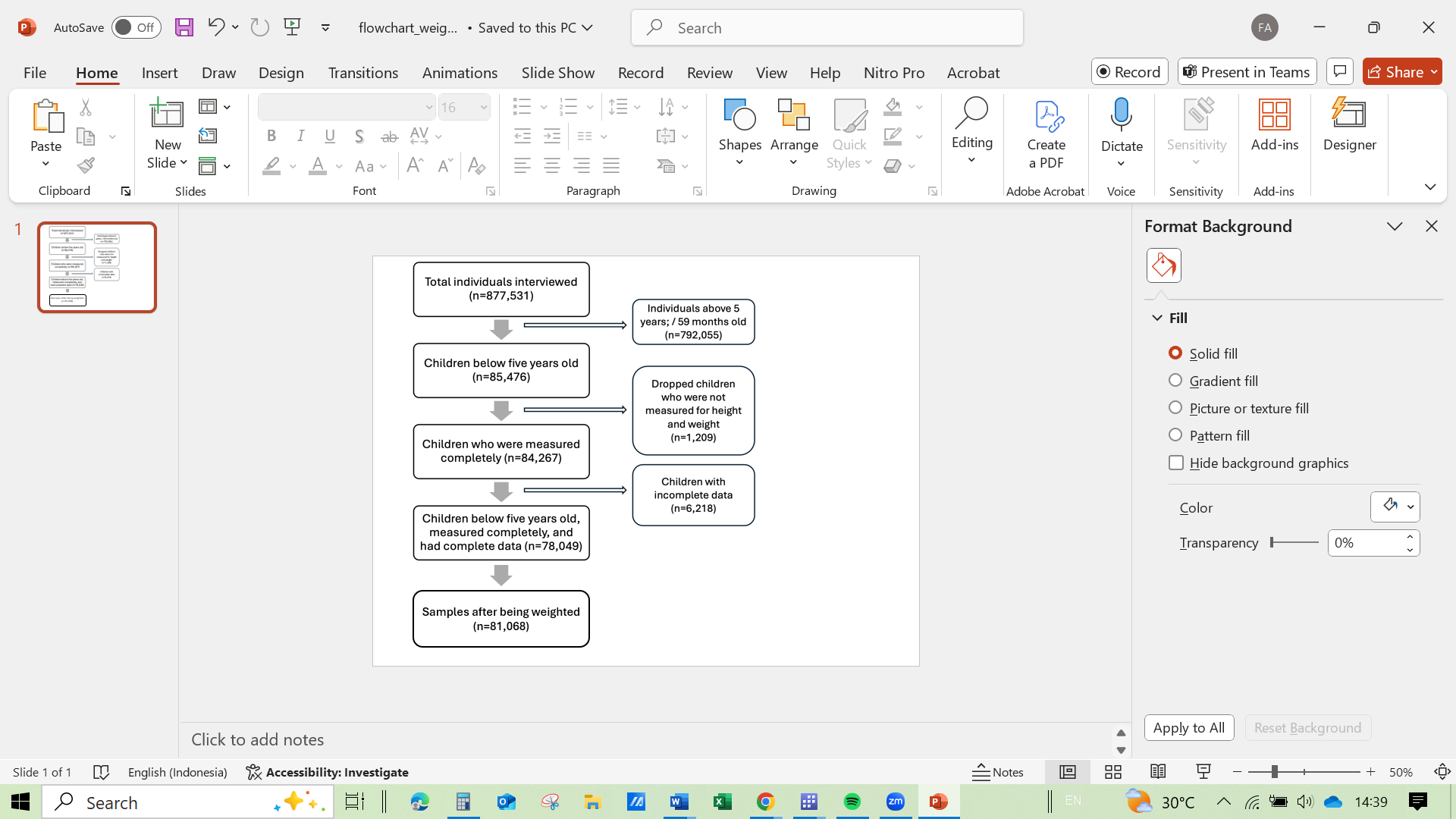

Supplement: Multimedia Appendix 1 [file pediatrics-v8-e68918-s001.docx]
